# Circulating Microparticles from Crohn’s Disease Patients Cause Endothelial and Vascular Dysfunctions

**DOI:** 10.1371/journal.pone.0073088

**Published:** 2013-09-03

**Authors:** Daniela Leonetti, Jean-Marie Reimund, Angela Tesse, Stéphanie Viennot, Maria Carmen Martinez, Anne-Laure Bretagne, Ramaroson Andriantsitohaina

**Affiliations:** 1 LUNAM Université, Angers, France; 2 INSERM, U1063, Angers, France; 3 Université de Caen Basse-Normandie, UFR de Médecine, EA 4652 (Laboratoire «Microenvironnement Cellulaire et Pathologies»), SF 4206 ICORE, Caen, France; 4 CHU de Caen, Service d’Hépato-Gastro-Entérologie et Nutrition, Pôle Médecine d’Organes et Cancérologie, Caen, France; University Medical Center Utrecht, Netherlands

## Abstract

**Background:**

Microparticles (MPs) are small vesicles released during cell activation or apoptosis. They are involved in coagulation, inflammation and vascular dysfunction in several diseases. We characterized circulating MPs from Crohn’s Disease (CD) patients and evaluated their effects on endothelial function and vascular reactivity after *in vivo* injection into mice.

**Methods:**

Circulating MPs and their cellular origins were examined by flow cytometry from blood samples from healthy subjects (HS) and inactive or active CD patients. MPs were intravenously injected into mice. After 24 hours, endothelial function and vascular reactivity were assessed.

**Results:**

Circulating MP levels did not differ between HS and inactive CD patients except for an increase in leukocyte-derived MPs in CD. Active CD patients compared to HS displayed increased total circulating MPs, pro-coagulant MPs and those from platelets, endothelium, erythrocytes, leukocytes, activated leukocytes and activated platelets. A significant correlation was found between total levels of MPs, those from platelets and endothelial cells, and the Harvey-Bradshaw clinical activity index. MPs from CD, but not from HS, impaired endothelium-dependent relaxation in mice aorta and flow-induced dilation in mice small mesenteric arteries, MPs from inactive CD patients being more effective than those from active patients. CDMPs induced vascular hypo-reactivity in aorta that was prevented by a nitric oxide (NO)-synthase inhibitor, and was associated with a subtle alteration of the balance between NO, reactive oxygen species and the release of COX metabolites.

**Conclusions:**

We provide evidence that MPs from CD patients significantly alter endothelial and vascular function and therefore, may play a role in CD pathophysiology, at least by contributing to uncontrolled vascular-dependent intestinal damage.

## Introduction

Crohn’s disease (CD) is a relapsing digestive inflammatory disorder whose causes remain still unknown. It is usually accepted that three main interacting actors contribute to its pathophysiology: a genetic susceptibility, environmental factors (especially smoking), and priming by the enteric flora (also called microbiota) [1]–[5]. As a result, inappropriate intestinal inflammatory and immune responses occur and lead to immune-mediated tissue injury and the subsequent clinical symptoms and signs of the disease. Beside this current pathophysiological hypothesis, several data indicate that CD is also associated with intestinal microvascular dysfunction [6], [7]. Early studies showed occlusion of the vessels supplying the mesenteric margin that might cause ischemia in CD inflamed terminal ileum [8]. Moreover, it has been observed that mesenteric vascular damage appears before inflammatory infiltration of the intestinal *lamina propria* [9]. Peripheral arterial mesenteric thrombosis and multifocal mesenteric ischemic infarction in distal small arteries at the mesenteric border, next to the mucosal lesions have also been reported in CD [10].

Among the direct consequences of these vascular alterations, the risk of peripheral mesenteric microthrombosis, endothelial dysfunction and modifications of vascular tone are well described [11], [12]. In submucosal arterioles isolated from areas of chronic intestinal inflammatory damage from inflammatory bowel disease (IBD) [CD and ulcerative colitis (UC)] patients, endothelial dysfunction has been shown to be associated with reduced nitric oxide (NO) and increased oxidative stress [13]. However, other authors described an unchanged endothelium-dependent and -independent relaxation in the mesenteric arteries from CD patients but a decrease of vascular tone [14]. In contrast to these results, we found no modification on vascular reactivity of small mesenteric arteries (SMA) from CD patients despite an increased release of proinflammatory cytokines from their intestinal mucosa. Indeed, we found that a balance between vasoconstrictor products from cyclo-oxygenase-2 (COX-2) and unidentified vasodilator products may explain that vascular reactivity remained unchanged [15].

Functional alterations in inflamed gut can result from over-production of different inflammatory mediators including not only NO and eicosanoids but also endotoxins and cytokines [16]. Other factors that may trigger these effects can be circulating microparticles (MPs) which are small membrane vesicles shed from cells in response to activation or apoptosis [17]–[21]. Present in blood of healthy subjects (HS), MPs have been studied in various diseases, like diabetes, metabolic syndrome, sepsis or obstructive sleep apnoea in which their number, cellular source or composition are altered [22]–[25]. MPs might play a major role in interactions with circulating cells or the components of the vessel wall. Indeed, they have been implicated in coagulation, inflammation and vascular function [19], [20], [26]–[28].

In active CD patients, increased circulating levels of pro-coagulant MPs that are reduced upon treatment with infliximab with no correlation to the clinical Van Hees activity index have been reported [29]. On the other hand, elevated MP levels derived from platelets correlated with CD Activity Index (CDAI) and activation of platelet aggregation evaluated by P-selectin expression. The level of platelet MPs decreased after remission of the disease [30].

To the best of our knowledge, studies analysing total circulating MPs and their cellular origins with clinical characteristics of CD patients has never been assessed in details. Moreover, the role of these MPs in the regulation of endothelial dysfunction and vascular reactivity in CD patients is not known. Thus, the aim of this work was to characterize circulating MPs from patients with CD according to their cellular origins and their correlation with clinical status. Then, MPs were injected intravenously into mice to test their effects on both endothelial function and vascular reactivity in conductance arteries and SMA.

## Materials and Methods

### Patients

Thirty nine adult patients (27 women, 12 men) with CD according to the Lennard-Jones criteria [31] were recruited at the Service d’Hépato-Gastro-Entérologie et Nutrition from the Caen University Hospital (CHU de Caen, France). Patients were considered for enrolment if they are referred to the Medical Centre for the treatment of active luminal CD, for maintenance treatment in inactive CD or for surveillance clinical visit if they were older than 18 years and if they had no exclusion criteria. Exclusion criteria were: current or recent (at least 1 month before) intestinal infection or infectious complication of CD, extra-digestive infection, or pregnancy. Clinical activity was determined using the Harvey-Bradshaw index [32]. This index allows an instantaneous calculation of disease activity using clinical items at the time of hospitalization or surveillance outpatients visit, by contrast to the CD Activity Index (CDAI) which needs the record of clinical symptoms during the 7 days before calculation of disease activity. A good correlation has been reported between the Harvey-Bradshaw index and the CDAI [32]. The clinical items recorded to determine the Harvey-Bradshaw index are: (i) number of liquid or soft stools per day, (ii) abdominal pain (none = 0, mild = 1, moderate = 2, severe = 3), (iii) general well-being (very well = 0, slightly below par = 1, poor = 2, very poor = 3, terrible = 5), (iv) presence of abdominal mass at clinical examination (non = 0, dubious = 1, definite = 2, definite and tender = 3), and (v) presence of the following complications (arthralgia, uveitis, erythema nodosum, aphtous ulcers in mouth, anal fissure, anal fistula, abscess; 1 point for each if present). Disease was considered inactive if the Harvey-Bradshaw index was <4, active if the Harvey-Bradshaw index was comprised between 4 and 12, active and severe when the Harvey-Bradshaw index was >12. Their median age was 31 years (extremes: 19–60). Clinical characteristics of patients were summarized in Table 1.

**Table 1:** Clinical characteristics of Crohn’s disease (CD) patients.

	Inactive CD patients (*n* = 13)	Active CD patients (*n* = 26)
Gender (women/men)	7/6	20/6
Age (median, extremes)	37 (19–59)	28 (19/60)
Disease activity: Harvey-Bradshaw index (HB) median (extremes)	HB: 1 (0–3)	HB: 10 (5–21); Non-severe: *n* = 15; HB: 9 (5–10); Severe: *n* = 11; HB: 14 (13–21)
Disease duration (months) (median, extremes)	108 (17–304)	84 (0.5–276)
Type of Crohn’s disease	Inflammatory: *n* = 9; Stenotic: *n* = 4	Inflammatory: *n* = 22; Stenotic: *n* = 4
Extension	Only ileal: *n* = 4; Only colonic: *n* = 4; Ileocolonic: *n* = 5	Only ileal: *n* = 5; Only colonic: *n* = 10; Ileocolonic: *n* = 11
Perineal disease (yes/no)	4/9	7/19
Treatment*	No treatment: *n* = 0; Corticosteroids: *n* = 7; Azathioprine:*n* = 3; Methotrexate: *n* = 1; Anti-TNF mAb**: *n* = 9	No treatment: *n* = 11; Corticosteroids: *n* = 8; Azathioprine: *n* = 7; Methotrexate: *n* = 2; Anti-TNF mAb**: *n* = 5
Previous surgery (ileocolonic resection)*** (yes/no)	2/11	2/24

* Total is higher than the number of patients as some of them receive combination of medication.

** mAb: Monoclonal antibody. All patients, except two in the active CD patients group failing adalimumab therapy at the time of inclusion, received infliximab.

*** Except ileocolonic resection no patient had underwent other type of small bowel and/or colonic surgery.

Biological parameters (as well as the Harvey-Bradshaw index) were also determined at the time of blood collection for MP isolation in CD patients (usually the day following their admission at hospital). These include total leukocytes, polymorphonuclear leukocytes (PMN), lymphocytes and platelet counts, haemoglobin level, C-reactive protein (CRP) (mg/L) and albumin (g/L) concentrations.

The control group consisted of 28 healthy subjects (named HS or controls: 13 women, 15 men) aged 25.5 years old (22–56; *p = *0.15 compared to the CD patients group). HS had no history of digestive or extra-digestive disease, had no familial history of digestive disorders (in particular history of IBD), and, except oral contraception in women, do not take any medication.

In accordance with the ethical guidelines of the Declaration of Helsinki and the ethical French guidelines, the local Ethics Committee [Comité de Protection des Personnes (CPP) Nord-Ouest III, Caen, France] approved the study. The Agence Française de Sécurité Sanitaire des Produits de Santé (AFSSAPS), a French regulatory health authority approved also the study. All subjects provided written informed consent.

### Blood Collection and Cell-derived Circulating MP Isolation

Blood samples from CD patients and HS (20 mL) were collected by sterile venous puncture in trisodium citrate glass tubes (Vacutainer, Becton Dickinson, Le Pont de Claix, France) at a final volume ratio of 9∶1. Blood collection was performed in the morning in overnight fasting patients and controls. The MP isolation procedure began less than 30 minutes after blood collection. Samples were centrifuged as previously described [23]–[25]. Briefly, samples were centrifuged for 20 minutes at 270 *g*, and plasma was then harvested and centrifuged at 1,500 *g* in order to obtain platelet-free plasma (PFP). Two hundred µL of PFP were immediately frozen and stored at −80°C until subsequent use. Then, remaining PFP was subjected to three series of centrifugations at 21,000 *g* for 90 minutes in order to eliminate plasma and to pellet MPs for the subsequent *in vivo* studies. Supernatant was replaced by 0.9% saline salt solution [23]–[25]. Finally, MP pellets were resuspended in 200 µL of 0.9% saline salt solution and stored at 4°C until subsequent use. Saline salt solution for the last supernatant was used as control (vehicle).

### Characterisation of MP Phenotype

MP subpopulations were characterised in PFP according to the expression of membrane-specific antigens by flow cytometry [23]–[25]. MPs derived from platelets, erythrocytes, leukocytes, endothelial cells and granulocytes were identified using anti-CD41, anti-CD235a, anti-CD45, anti-CD146 and anti-CD66b antibodies, respectively. Anti-CD62P and anti-CD62L antibodies were used to identify P-selectin-positive and L-selectin-positive MPs, respectively. Irrelevant human IgG was used as an isotype-matched negative control for each sample. Annexin-V (Beckman Coulter, Villepinte, France) binding was used to numerate phosphatidylserine-expressing MPs (2 µL of annexin-V/5 µL PFP). To determine MPs concentrations, 10 µL of PFP were incubated with 5 µL of specific antibody (Beckman Coulter). After 45 minutes of incubation, samples were diluted in 300 µL of 0.9% saline salt solution or annexin-V labelling buffer, respectively. Then, an equal volume of sample and Flow-count fluorospheres (Beckman Coulter) were added in order to calculate MP concentration The Flow-count fluorospheres were used to quantify MPs. Each lot of Flow-count fluorospheres has a specific concentration of fluorospheres. When identical volumes of a sample and Flow-count fluorospheres are used, a ratio of MPs in the sample to fluorospheres is established. Since the concentration of fluorospheres is known, the absolute count of the MPs can be determined using the MXP software (Beckman Coulter). The samples were analysed in a flow cytometer 500 MPL System (Beckman Coulter). Sample analysis was stopped after the count of 10,000 events.

### Vascular Reactivity

All animal studies were performed using approved institutional protocols. The protocol was approved by The Committee on the Ethics of Animal Experiments of CEEA Pays de la Loire (Permit Number: CEEA.2009.9). MPs were injected into the tail vein in male Swiss mice (6–8 week old) at the circulating level of MPs detected in the blood of CD patients (CDMPs) and HS (HSMPs) or with saline salt solution (vehicle). These values ranged from 1,642 to 63,184 MPs/µL of plasma for healthy subjects and 1,396 to 18,944 MPs/µL of plasma for CD patients, indicating that several healthy subjects had circulating levels of MPs greater than several CD patients. Using MPs from other pathologies, we have previously shown that the duration of 24 hours treatment with MPs induced changes of vascular function. Furthermore, the injection of MPs from human subjects in mice for 24 hours did not induce immunological reactions under the same experimental conditions [23]–[25]. Non-invasive blood pressure was measured by tail-cuff method (Letica, Barcelona, Spain) in animals before and 24 hours after MP injection. Animals were trained everyday over a period of a week to get accustomed to the device. A total of 10 consecutive readings of systolic pressure and heart rate were daily recorded and averaged. Twenty-four hours after having been injected, mice were sacrificed and aortic rings and SMA were isolated and segments (2 mm-long) were mounted on a wire myograph and arteriograph for measurement of vascular reactivity. The functionality of the endothelium was assessed by cumulative application of acetylcholine (1 nmol/L-10 µmol/L, Sigma-Aldrich, St. Quentin, Fallavier, France) in aortas precontracted with U46619 (0.1 µmol/L, Sigma-Aldrich).

SMA (100 to 130 µm in diameter) were mounted in a video-monitored perfusion system (Living Systems Instrumentation, Burlington, VT) to study the physiological endothelial dilatation in response to shear stress. Diameter changes were measured by increasing flow rate (0 to 92 µL/min) under a constant intraluminal pressure of 75 mm Hg. The integrity of the endothelium was studied by using acetylcholine (10 µmol/L) in the arteries pre-contracted with U46619 (1 µmol/L). The different components of the dilation were determined by using the following inhibitors: the NO-synthase inhibitor *N* ^G^-nitro-L-arginine (L-NA, 100 µmol/L; Sigma Aldrich), the non-selective COX inhibitor indomethacin (10 µmol/L; Sigma Aldrich). The NO, COX and the endothelial-derived hyperpolarizing factor (EDHF) component of the response of dilation were calculated as previously described [25]. Vascular reactivity was evaluated by cumulative application of serotonin (5-HT, 3 nmol/L-10 µmol/L; Sigma Aldrich) to vessels with functional endothelium in the absence or presence of the given inhibitor: L-NA (100 µmol/L), indomethacin (10 µmol/L), the selective COX-1 inhibitor 5-(4-Chlorophenyl)-1-(4-methoxyphenyl)-3-trifluoromethyl pyrazole (SC-560 10, µmol/L) and the selective COX-2 inhibitor *N*-(2-cyclohexyloxy-4-nitrophenyl) methanesulfonamide (NS-398, 10 µmol/L; Sigma Aldrich). All inhibitors were used at maximal active concentrations as reported in many of our previous studies [24], [25], [27].

### NO Spin Trapping and Electronic Paramagnetic Resonance (EPR) Studies

The detection of NO production was performed using the technique with Fe^2+^ diethyldithiocarbamate (DETC, Sigma Aldrich) as spin trap. Isolated aortas from mice injected respectively with CDMPs, HSMPs and vehicle were incubated for 45 minutes in Krebs-Hepes buffer (BSA (20.5 g/L), CaCl_2_ (3 mmol/L) and L-arginine (0.8 mmol/L)) and after treated with 250 µL of colloid Fe(DETC)_2_ and incubated at 37°C for 45 minutes [24], [33]. The organs were immediately frozen in plastic tubes. NO measurements were performed on a tabletop x-band spectrometer miniscope (MS200; Magnettech, Berlin, Germany). Values are expressed as amplitude of signal per weight of dried tissue.

### Superoxide Anion (O_2_^−^) Determination by EPR

The aortas isolated from mice injected with CDMPs, HSMPs or vehicle were dissected and allowed to equilibrate in deferoxamine-chelated Krebs-Hepes solution containing 1-hydroxy-3-methoxycarbonyl-2,2,5,5-tetramethylpyrrolidin (CMH; Noxygen, Mainz, Germany) (500 µmol/L), deferoxamine (25 µmol/L), and DETC (5 µmol/L) under constant temperature (37°C) for 45 minutes. The reaction was stopped by putting samples on ice. The organs were frozen in plastic tubes and analyzed in a Dewar flask by EPR spectroscopy [24]. Values are expressed as amplitude of signal per weight of dried tissue.

### Western Blotting

The aortas isolated from mice injected respectively with vehicle, HSMPs and active CDMPs were dissected and homogenized for protein extraction. Proteins (40 µg) were separated using 10% sodium dodecyl sulphate-polyacrylamide gel electrophoresis. Blots were probed with anti-inducible NOS (iNOS) (BD Bioscences, San Jose, CA). A polyclonal rabbit anti-mouse β-actin antibody (Sigma Aldrich) was used as a loading control. Membranes were washed at least three times in Tris-buffer solution containing 0.05% Tween and incubated for 1 h at room temperature with the horseradish peroxidase (HRP)-conjugated secondary antibody (Amersham, Piscataway, NJ). Protein bands were detected by SuperSignal West Femto Maximum Sensitivity Substrate (Pierce, Rockford, IL) according to the protocol of the manufacturer.

### Data Analysis

Data are represented as mean ± SEM, *n* represents the number of patients or mice. In mouse aorta studies, each condition was performed by duplicate (2 rings from the same mouse). Statistical analyses were performed by a one-way analysis of variance and Mann- Whitney *U*-test for data analysis between groups or two-way analysis of variance for repeated measures and subsequent Bonferroni post hoc test. *P*<0.05 was considered to be statistically significant.

## Results

### Patient Characteristics

Clinical characteristic of CD patients are summarized on Table 1. Twenty six patients had active CD with a median Harvey-Bradshaw index of 10 (5–21), among which 15 were considered as having active non-severe disease and 11 patients presenting severe disease. Thirteen CD patients had inactive disease (median Harvey-Bradshaw index: 1 (0–3)).

Neither age nor disease extension were statistically different between inactive and active CD patients. CD patients received infliximab (*n* = 12), adalimumab (*n* = 2), azathioprine (*n* = 10), methotrexate (*n* = 3) or oral prednisone (*n* = 12), or oral budesonide (*n* = 3).

CRP concentration was significantly higher in active CD patients, whereas albumin concentration was significantly lower compared to inactive CD patients. Total leukocytes, PMN, and lymphocytes were not different between active and inactive CD patients. Platelet count was significantly increased in active CD patients whereas haemoglobin was decreased (Table 2). Finally, Harvey-Bradshaw index was positively correlated to CRP concentration (r’ = 0.70, *p*<0.0001) and negatively to albumin concentration (r’ = −0.52, *p = *0.042). CRP and albumin concentrations were negatively correlated to each other (r’ = −0.67, *p = *0.0002).

**Table 2:** Biological parameters of inactive and active Crohn’s disease (CD) patients.

	Inactive CD patients (*n* = 13)	Active CD patients (*n* = 26)
C-reactive protein (CRP) (mg/L)	2.90±0.75	47.22±11.58***
Albumin (g/L)	39.07±1.26	32.28±1.55**
Total leukocytes (number/mm^3^)	8,140±971	8,256±725
Polymorphonuclear leukocytes (number/mm^3^)	5,458±956	4,859±531
Lymphocytes (number/mm^3^)	2,125±337	2,253±208
Platelets (number/mm^3^)	271,385±21,614	435,286±33,651*
Hemoglobin (g/dL)	13.3±0.3	11.6±0.5*

* *p*<0.05,

** *p*<0.01,

*** *p*<0.001 vs inactive CD patients.

### Circulating MPs and their Cellular Origin

**Figure 1:**
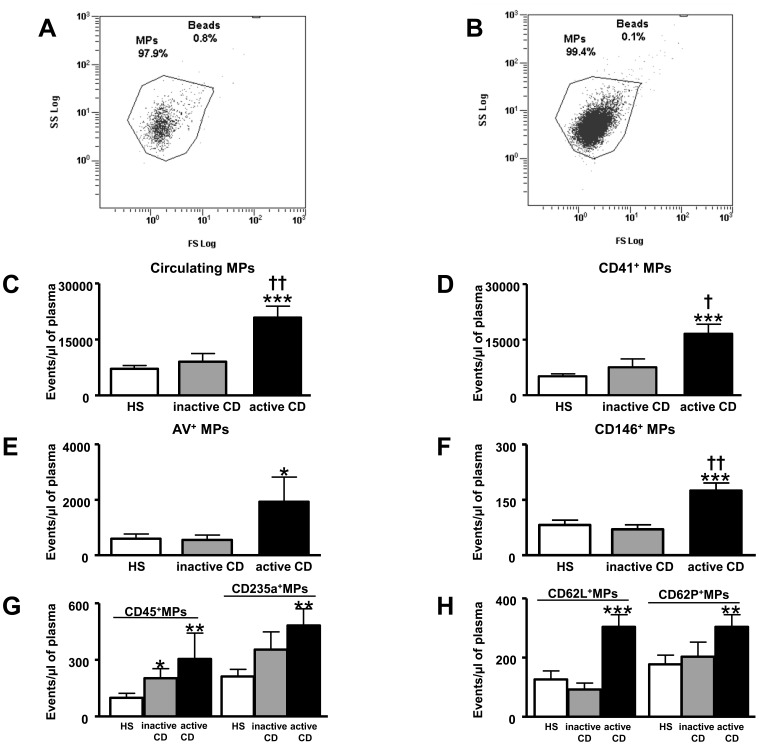
Circulating microparticles (MPs) from healthy subjects (HS) (A) and Crohn’s disease (CD) patients (B) and Flowcount beads (Beads region, 10 µm diameter) are visualized in a side scatter (SS)/forward scatter (FS) logarithmic representation. MPs are defined as events with size 0.1 to 1 µm gated in the “MPs” window. Circulating MP levels in patients with inactive CD and active CD compared to HS. Total circulating MPs (**C**), CD41^+^ MPs (**D**), annexin V^+^ MPs (**E**), CD146^+^ MPs (**F**), CD45^+^ and CD235a^+^ MPs (**G**), CD62L^+^ and CD62P^+^ MPs (**H**). MPs from HS (*n* = 28), MPs from inactive CD patients (inactive CD *n* = 13) and MPs from active CD patients (active CD *n* = 26). Results are expressed as events/µL of plasma and given as mean ± SEM. *p<0.05, ***p<0.001 *vs* HS; †*p*<0.05, ††*p*<0.01 active CD *vs* inactive CD.

The total number of circulating MPs was significantly increased in active CD patients compared with HS and inactive CD patients (Figure 1C). Phenotypical characterisation of cellular origin of MPs from HS and inactive CD patients did not show significant difference between the two groups except that of leukocytes (CD45^+^) MPs (Figure 1D–1H). Interestingly, compared to HS, active CD patients displayed increases of pro-coagulant (annexing V^+^) MPs and those from platelets (CD41^+^), endothelial cells (CD146^+^), leukocytes (CD45^+^), erythrocytes (CD235a^+^), activated leukocytes (CD62L^+^) and activated platelets (CD62P^+^) (Figure 1D–1H). MPs from macrophages (CD11b^+^) and granulocytes (CD66b^+^) were not significantly different between HS and active CD patients (not shown). Finally, active CD patients showed increased total circulating MPs, platelet- and endothelial-derived MPs when compared to inactive patients (Figure 1C, 1D and 1F).

### Relation between MP Levels and Clinical Characteristics

**Figure 2:**
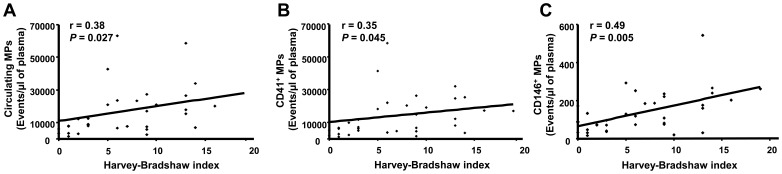
Correlations between microparticle (MP) levels and disease activity. Correlations between total circulating MPs, CD41^+^ MPs, CD146^+^ MPs and Harvey-Bradshaw index (**A**–**C**).

Total MP concentration was not correlated to disease duration (r’ = 0.104, *p = *0.55). Correlation analysis for disease activity showed that total circulating MPs and those from platelets and endothelial cells were positively correlated to the Harvey-Bradshaw index (Figure 2A–2C).

Among biological data, total circulating MPs, pro-coagulant, platelet-, endothelial- and erythrocyte MPs were correlated to CRP. Only a trend of correlation was observed for activated leukocyte MPs. There was no correlation between total MPs with albumin concentration, haemoglobin level, leukocytes, PMN or lymphocyte counts, although there was a trend with platelet count. Endothelial-derived MPs were negatively correlated to albumin concentration (Table 3). There was no correlation between the levels of macrophage-, leukocyte-, granulocyte- and activated platelet-MPs with other measured biological characteristics of CD patients (data not shown).

**Table 3:** Correlations between circulating MPs level and biological parameters of CD patients.

	Circulating MPs	AV^+^ MPs	CD41^+^ MPs	CD146^+^ MPs	CD235^+^ MPs	CD62L^+^ MPs
C-reactive protein (CRP) (mg/L)	r’ = 0.56; *p* * = *0.0014	r’ = 0.44; *p* = 0.011	r’ = 0.51; *p* = 0.0032	r’ = 0.67; *p* = 0.0001	r’ = 0.36; *p* = 0.039	r’ = 0.33; *p* = 0.056
Albumin (g/L)	r’ = − 0.17; *p* = 0.35	r’ = − 0.14; *p* = 0.45	r’ = − 0.115; *p* = 0.53	r’ = − 0.037; *p* = 0.05	r’ = − 0.21; *p* = 0.82	r’ = − 0.94; *p* = 0.61
Platelets (number/mm^3^)	r’ = 0.34; *p* = 0.058	r’ = − 0.55; *p* = 0.76	r’ = 0.34; *p* = 0.06	r’ = 0.165; *p* = 0.36	r’ = 0.056; *p* = 0.75	r’ = 0.27; *p* = 0.14

Neither total MPs nor their cellular origins were significantly different considering disease extension despite a trend to a decrease in ileal compared to colonic or ileocolonic CD for CD62P^+^ MP levels (*p = *0.0723).

The type of disease (“stenotic”, “inflammatory”) did not influence MP levels. Patients with perineal disease showed lower levels of CD66b^+^ MPs (*p = *0.0207) and a trend to lower CD235a^+^ MPs (*p = *0.074).

### MPs from CD Induce Endothelial Dysfunction in Aorta and SMA

**Figure 3:**
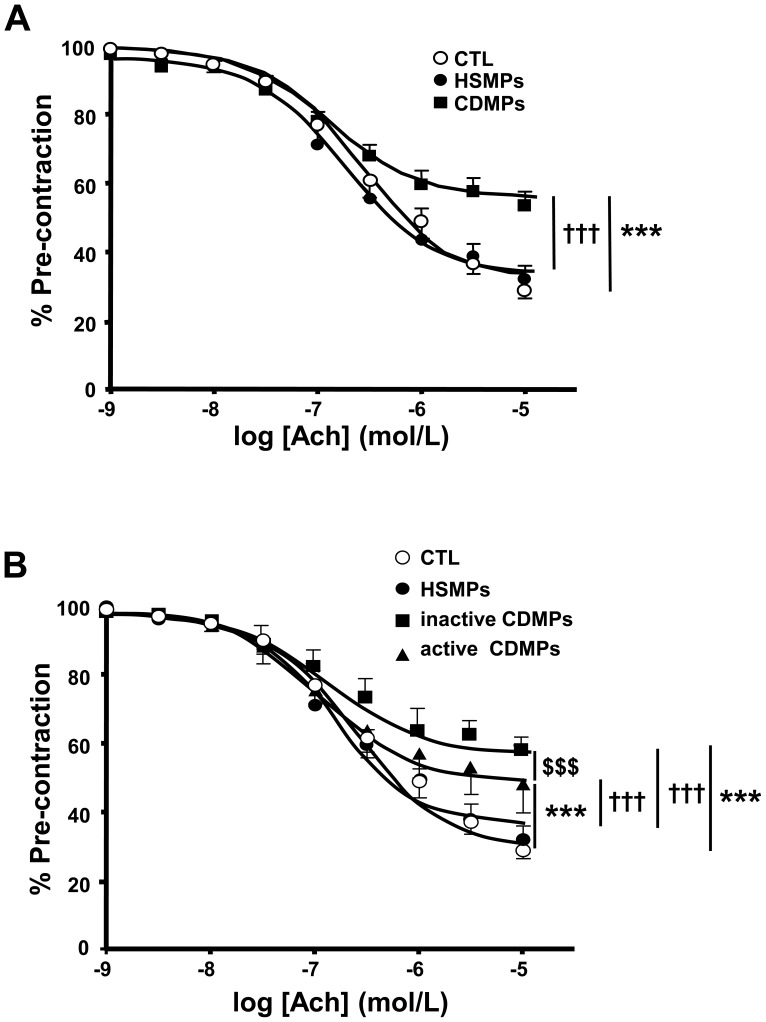
Microparticles (MPs) from CD patients impair endothelium-dependent relaxation in mouse aorta. Acetylcholine (Ach)-induced relaxation in aortic rings isolated from mice injected *in vivo* with vehicle (controls (CTL)), MPs from HS (HSMPs) and MPs from CD patients (CDMPs) (**A**) subsequently separated in inactive CD patients (inactive CDMPs) (**B**) and active CD patients (active CDMPs) (**B**). Results are expressed as a percentage of U46619-induced pre-contraction and given as mean ± SEM (*n* = 5–11). ***p<0.001 *vs* CTL; ^†††^ *p*<0.001 *vs* HSMPs; ^$$$^ *p*<0.001 active CDMPs *vs* inactive CDMPs.

The endothelium-dependent relaxation to acetylcholine was significantly impaired in aorta isolated from mice injected with CDMPs compared to aorta isolated from mice injected either with vehicle or with HSMPs (E*max*: 69.3±2.1%, 65.2±2% and 45.1±1.02% for CTL, HSMPS and CDMPs, respectively *p*<0.001) (Figure 3A). Injection of either inactive or active CDMPs reduced acetylcholine-induced aortic endothelium-dependent relaxation when compared to those taken from HSMP-treated mice. Unexpectedly, inactive CDMPs were more effective than active CDMPs in impairing endothelial function (E*max* inactive CDMPs 41.2±1.3%; E*max* active CDMPs 49.8±1.03%; *p*<0.01) (Figure 3B).

**Figure 4:**
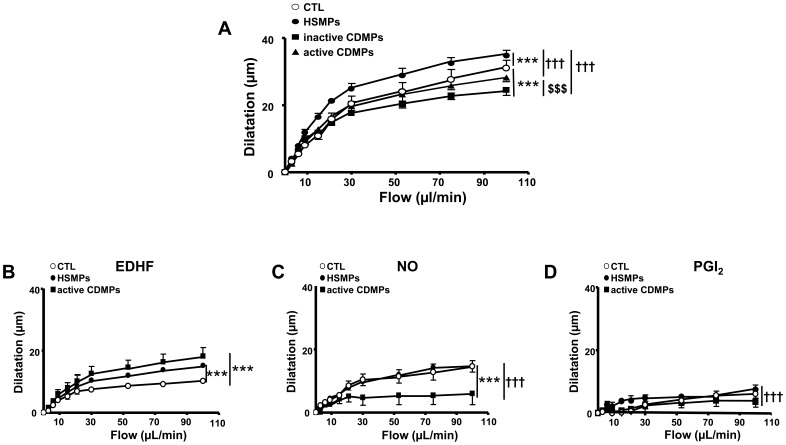
Microparticles (MPs) from CD patients impair flow-induced dilation in small mesenteric arteries. Flow-induced dilatation obtained in small mesenteric arteries isolated from mice injected *in vivo* with vehicle (controls (CTL)), MPs from HS (HSMPs) and MPs from inactive CD patients (inactive CDMPs) and active CD patients (active CDMPs) (**A**). Dilation expressed in µm in response to flow expressed in µL/min: dilation dependent to endothelium-derived hyperpolarizing factor (EDHF) (**B**), to nitric oxide (NO) (**D**), and to cyclooxygenase products prostaglandin (PGI_2_) (**D**). Results are given as mean ± SEM (*n* = 5–10). ***p<0.001 *vs* CTL; ^†††^ *p*<0.001 *vs* HSMPs; ^$$$^ *p*<0.001 active CDMPs *vs* inactive CDMPs.

In SMA, HSMPs significantly enhanced flow-induced dilation when compared to control (Figure 4A), due to increased EDHF-component of the response (Figure 4B). Compared to control, inactive CDMPs (*p*<0.001) but not active CDMPs reduced flow-induced dilation (Figure 4A), indicating that, as for the aorta, active CDMPs are less effective in eliciting endothelial dysfunction compared to inactive CDMPs (Figure 4A). This reduced efficiency of active CDMPs to decrease flow-induced dilation resulted in the decrease of NO-component (Figure 4C) compensated by the increase of EDHF-component (Figure 4B) without affecting prostacyclin-component (Figure 4D) of the response.

### CDMPs Induce Ex Vivo Vascular Hypo-reactivity in Mouse Aorta

**Figure 5:**
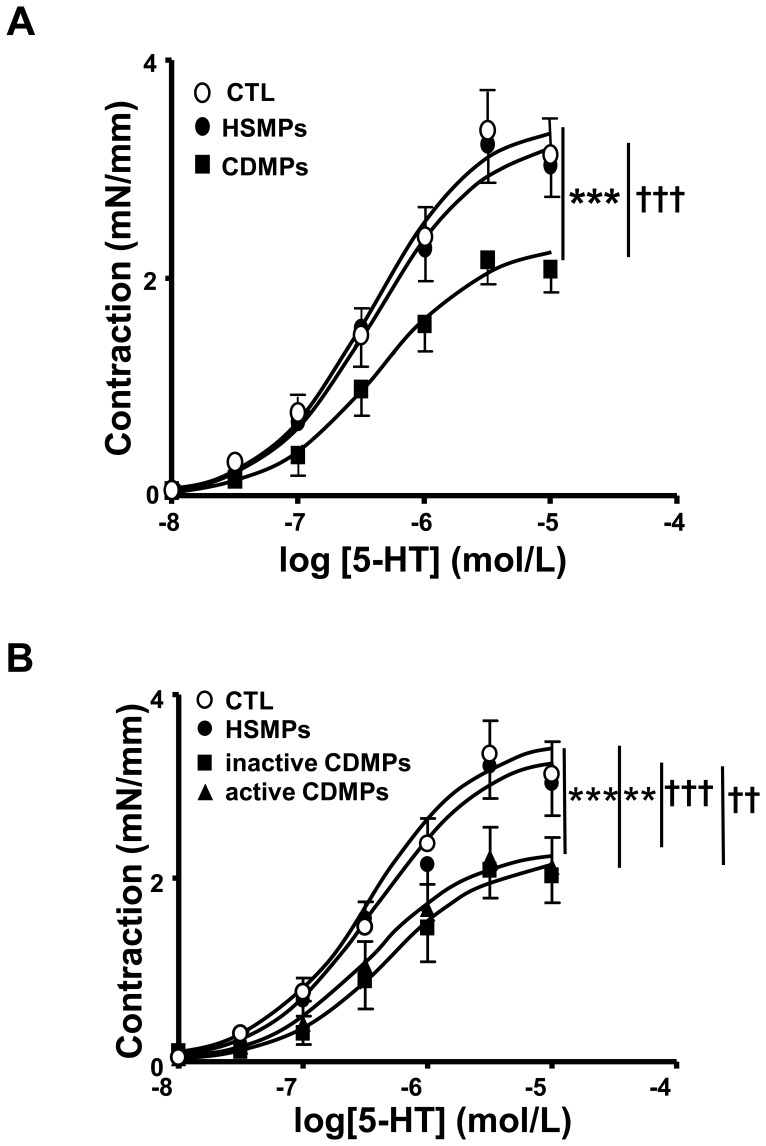
Microparticles (MPs) from CD patients decrease 5-HT contraction in mouse aorta. Concentration-effect curves in response to 5-HT in aortic rings isolated from mice injected *in vivo* with vehicle (controls (CTL)), MPs from HS (HSMPs) and MPs from CD patients (CDMPs) (**A**) subsequently separated in inactive CD patients (inactive CDMPs) (**B**) and active CD patients (active CDMPs) (**B**). Contraction are expressed in mN/mm. Results are given as mean ± SEM (*n* = 6–13). **p<0.01, ***p<0.001 *vs* CTL; ^††^ *p*<0.001, ^†††^ *p*<0.001 *vs* HSMPs.

5-HT produced a concentration-dependent increase in tension in aortic rings from all groups of mice; however, vascular reactivity to the agonist was markedly decreased in mice treated with CDMPs compared to those treated either with vehicle or HSMPs (Figure 5A). Both inactive and active CDMPs reduced vascular reactivity to 5-HT to the same extent when compared to vehicle or HSMPs (Figure 5B). Since no difference of hyporeactivity was observed between inactive and active CD patients, the mechanisms involved for this process was only investigated in vessels from mice treated with active CDMPs.

### Involvement of NO and O_2_^−^ in Active CDMP-induced Vascular Hypo-reactivity

**Figure 6:**
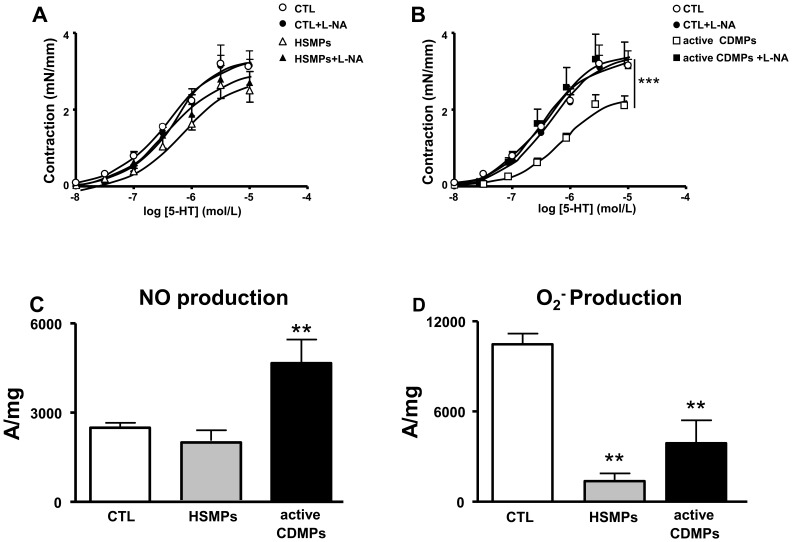
Microparticles (MPs) from active CD patients induce nitric oxide (NO) overproduction in mouse aorta. Concentration-effect curves of 5-HT in the presence and in the absence of L-NA in aortic rings isolated from mice injected *in vivo* with vehicle (controls (CTL)) and MPs from HS (HSMPs) (**A**) and with MPs from active CD patients (active CDMPs) (**B**). Contraction are expressed in mN/mm. Results are given as mean ± SEM (*n* = 7–8). NO production (**C**) assessed by the amplitude of NO-Fe(DETC)_2_ complex signal and superoxide anion (O_2_^−^) production (**D**) assessed by the amplitude of O_2_^–^CMH complex signal in unit/weight in aorta from mice injected *in vivo* with vehicle (CTL), HSMPs and active CDMPs. Results are given as mean ± SEM (*n* = 5–7). **p<0.01; ***p<0.001 *vs* CTL.

To investigate the role of NO, the effect of the NO-synthase inhibitor, L-NA, was studied on the response to 5-HT. Interestingly, we found that inhibition of the NO pathway did not affect response to 5-HT in vessels from HSMPs (Figure 6A) but completely prevented the vascular hypo-reactivity induced by active CDMPs (Figure 6B). Direct *in situ* measurements of NO production were performed by EPR spectroscopy using Fe(DETC)_2_ as a spin trap. Aortas from vehicle, HSMP- and CDMP-treated mice, exhibited an EPR feature of signals derived from NO-Fe(DETC)_2_, being greater in aortas from active CDMP-treated mice than in vehicle- and HSMP-treated mice (Figure 6C).

Unexpectedly, vascular hypo-reactivity to 5-HT by active CDMPs was not associated with increased O_2_^−^ production. In contrast, both HSMPs and active CDMPs reduced O_2_^−^ production compared to control (Figure 6D).

### CDMPs Increase the Expression of iNOS in Aorta

**Figure 7:**
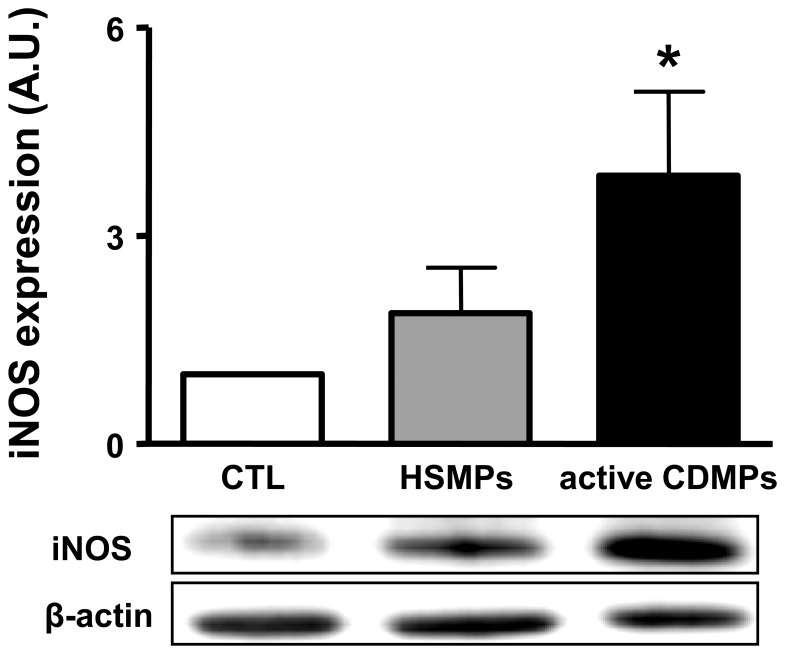
MPs from active CD patients increase the expression of iNOS in aorta. The aortas were isolated from mice injected with vehicle (CTL), MPs from healthy subjects (HSMPs) or with MPs from active CD patients (CDMPs). Western blotting was probed using antibodies raised against iNOS. Immunoblots were quantified by densitometric analysis. Data are representative of five separate blots, and the densitometry values are expressed in arbitrary units (A.U.) as mean ± SEM. *P<0.05 versus CTL.

In order to establish the source of NO, Western blots of aorta of mice treated with vehicle, HSMPs or CDMPs, were performed. As shown in Figure 7, MPs from CD patients, but not from HS patients, markedly enhanced iNOS expression that may explain the source of increased NO production.

### Involvement of COX Metabolites in Active CDMP-induced Vascular Hypo-reactivity

To investigate the role of COX metabolites in 5-HT-induced vasoreactivity, the effects of a non-selective inhibitor of COX (indomethacin) and a selective inhibitor of either COX-1 (SC-560) or COX-2 (NS398) were examined.

**Figure 8:**
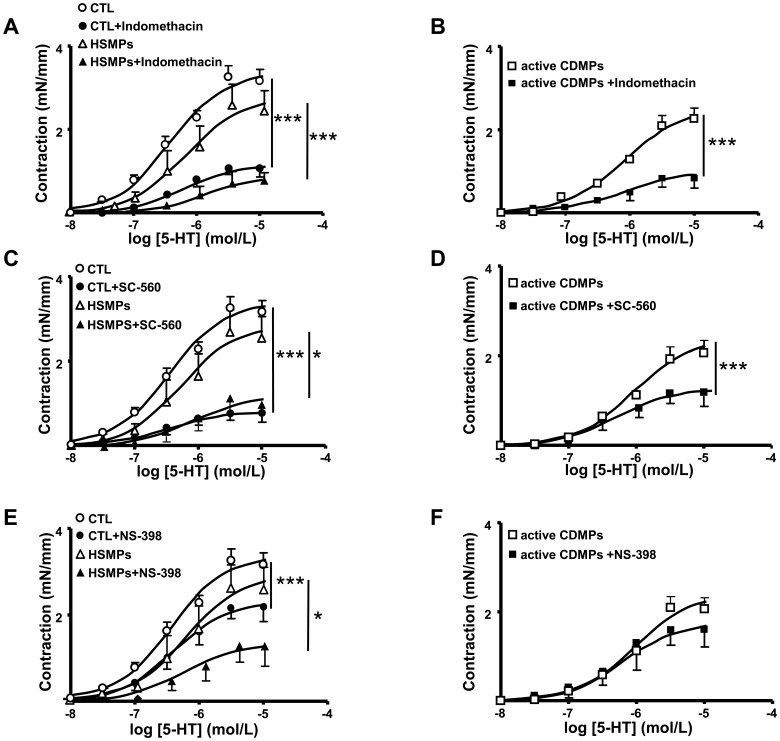
Involvement of COX-1 and COX-2 products in vascular reactivity of mouse aorta. Concentration-effect curves of 5-HT in the presence and in the absence of indomethacin (**A**, **B**), SC-560 (**C, D**) and NS-398 (**E, F**) in aortic rings isolated from mice injected *in vivo* with vehicle (controls (CTL)) (**A**, **C**, **E**), MPs from HS (HSMPs) (**A**, **C**, **E**) and MPs from active CD patients (active CDMPs) (**B**, **D**, **F**). Contraction are expressed in mN/mm. Results are given as mean ± SEM (*n* = 5–7). *p<0.05, ***p<0.001 *vs* control aortic rings.

In the presence either of indomethacin or SC-560, contractile response to 5-HT was reduced in aorta from all groups of mice (Figure 8A–8D). Meanwhile, vascular hypo-reactivity to 5-HT was still present in aortas from mice treated with CDMPs compared to HSMPs or vehicle. These results highlight that vasoconstrictor metabolite(s) sensitive to indomethacin and SC-560 participate in 5-HT-induced contraction, independently of the MP treatment. When COX-2 was specifically silenced using NS398, the response to 5-HT was impaired in vessels from vehicle and HSMP-injected mice (Figure 8E), suggesting the contribution of COX-2-derived vasoconstrictor metabolites. In contrast, blockade of COX-2 did not modify the response induced by active CDMPs (Figure 8F).

## Discussion

The present study reports an increased level of circulating MPs during the active but not inactive state of CD compared to HS, and detailed the effects of CDMPs on endothelial and vascular function. Analysis of cellular origin of MPs showed that only MPs from leukocytes were increased in inactive CD patients whereas active CD patients displayed increased circulating levels of MPs from platelets, pro-coagulant, endothelial cells, erythrocytes, leukocytes, activated leukocytes and activated platelets. Interestingly, a significant correlation was found between total levels of MPs, those from platelets and endothelial cells, and Harvey-Bradshaw index.

One of the most important messages of the present study is to provide evidence of pathophysiological relevance of MPs in CD as they strongly alter endothelial and vascular functions. As previously described for other pathologies [23]–[25], MPs were injected at the circulating level of MPs detected in the blood of CD patients. We observed that several HS patients had circulating levels of MPs greater than several CD patients, and even in this case, only MPs from CD patients induced changes in vasomotricity. Thus, the effects observed cannot be related to the different concentrations of circulating MPs in both groups of subjects, but rather to the composition of MPs. Despite the important discoveries related to CD pathophysiology reported during the last two decades in the fields of genetics, immunology or role of intestinal microbiota, vascular alterations may participate in CD lesions. Early observations came from histopathological reports showing clearly that anatomical lesions and distal vascular dysfunction were associated to CD (onset and/or more probably chronic course) [6]–[10]. Nevertheless, until now, the question remained to identify at least one factor explaining these abnormalities.

The present study provides evidence that CDMPs impaired endothelium-dependent relaxation in response to acetylcholine in aorta and the endothelial response to flow in SMA (MPs from inactive CD patients being more deleterious than those from active CD patients, a fact still remaining to be explained). These data contribute to explain – at least in part – several mechanisms participating in CD pathophysiology, especially in the long-term course of the disease. As shown, due to their impact on vascular function, MPs probably contribute to mesenteric thrombosis and multifocal mesenteric ischemic infarction in distal SMA. They may also induce endothelial dysfunction that is the primary event leading to the failure of vasoactive, anti-coagulant and anti-inflammatory effects of healthy endothelium. These data strongly suggest that CDMPs are able to induce *ex vivo* endothelial dysfunction and demonstrate one of their pathophysiological relevance in CD. These data are in accordance with our former works with MPs isolated from patients with preeclampsia, metabolic syndrome and obstructive sleep apnoea [23], [25], [27], [34]. Unexpectedly, MPs from inactive CD patients were more deleterious than those from active CD patients. Thus, firstly endothelial dysfunction still occurs during the inactive state. Secondly, one can advance the hypothesis of a potential attempt by the organism in the active phase of disease to partially offset these deleterious effects of MPs towards the endothelium. Indeed in SMA, the reduced NO-component of flow-induced vasodilatation was compensated by an increased EDHF-component of the response in vessels taken from mice treated with MPs from active CD patients. Thirdly, it is possible that the deleterious effects of leukocyte-derived MPs are counterbalanced, at least partially, by the other types of MPs (from platelets, erythrocytes, endothelial cells and those expressing L-selectin and P-selectin) (see below). These results may provide new information about the impact of disease activity on endothelial dysfunction observed in CD patients. Indeed, the results obtained in literature are conflicting. On the one hand, it has been reported elevated levels of asymmetric dimethylarginine, an endothelial dysfunction marker, in the serum of CD patients and these increased levels are positively correlated with the disease activity [35]. On the other hand, Roifman and coworkers did not observe the relationship between disease duration or clinical disease activity and microvascular endothelial dysfunction directly assessed in CD patients [36].

Interestingly, CDMPs promoted vascular hypo-reactivity by inducing the production of NO, decreasing O_2_^−^ content and altering the release of COX metabolites especially those from COX-2. The consequence of these alterations probably results in distal mesenteric vascular collapse, subsequent SMA occlusion, and finally the resulting local inflammation and tissue injury as observed in CD patients with active disease (or inactive disease; despite there were no macroscopic lesions in these patients, mucosal biochemical inflammation has been shown to be still present [16]). Indeed, MPs from both inactive and active CD patients are able to promote vascular hyporeactivity in response to 5-HT in mouse aorta. The fact that i.v. injection of MPs did not decrease systolic blood pressure (not shown) suggests that other factors are able to counteract the *in vivo* effects of MPs on systemic blood pressure or MPs might be involved in local inflammation and tissue injury only. These findings are in line with our previous studies on other disease such as preeclampsia, diabetes and metabolic syndrome [33], [34], [37]. In these pathologies, MPs induce vascular hyporeactivity by a mechanism that involves NO and COX pathways through enhanced expression of iNOS and COX-2 with subsequent increased NO and prostacyclin production, respectively. Altogether, these results suggest that CDMPs are able to act on smooth muscle and induce the release of vasodilator factors, at least NO without modification of oxidative stress, or alternatively, CDMPs evoke alteration of the balance between vasoconstrictor and/or relaxant factors (COX-2 metabolites) in smooth muscle cells. In the present study, we show that CDMPs induce an increase of iNOS expression accounting for the increase of NO production that explains the capacity of CDMPs to prevent the vasoconstriction. The excessive NO production observed in vascular tissue after administration of CDMPs, might suggest a deleterious effect by inducing nitration in tissues. Indeed, NO may interact with superoxide anion and produce peroxynitrite which can nitrate proteins. However, no effect on nitration was observed between the three groups of mice (not shown) indicating that NO affect endothelial relaxation and vascular reactivity by other mechanisms than protein nitration. In line with our results, we have reported in SMA isolated from inflamed CD colon a marked iNOS and COX-2 expressions and a balance between vasoconstrictor products from COX-2 and unknown vasodilator products that maintained vascular reactivity in a physiological range [15]. In the present study, we highlight the fact that MPs from CD patients play a significant role in vascular dysfunction including reduced response to vasoconstrictor agents. Thus, CDMPs could be considered as important mediators of inflammatory process that characterizes CD.

Platelet and endothelial MPs probably participate in the severity of the disease. With respect to increased platelet MPs, our data are in accordance with those reported in the literature using a new tool in the evaluation of thrombotic and bleeding disorders, the endogenous thrombin potential (ETP), the increase of which is related to the activity of this disease [38]. Very recently, it has been also reported an increased pro-coagulant function of MPs in pediatric patients in both active and quiescent CD compared with HS patients [39]. Regarding the involvement of endothelial MPs, an increased level of this type of MPs has been observed in several pro-inflammatory and prothrombotic pathological states such as pulmonary hypertension, venous thromboembolism and acute coronary syndrome [40]–[42]. Furthermore, endothelial MPs play a role in mechanism of coagulation, inflammation and angiogenesis [43]. Thus, the combination of these populations of MPs may participate in the inflammatory process inasmuch they can induce neutrophil and endothelial activation, monocyte adhesion, and recruitment of different inflammatory cells [18], [20]. However, the fact that the sole difference between HS and inactive CD patients regarding the type of MPs is the increase of the leukocyte-derived MPs in inactive CD patients suggest, on the one hand, that, during CD, both activation of leukocytes and MP release from these cells are enhanced, and on the other hand that leukocyte-derived MPs support, at least partially, the deleterious effect of MPs.

Limitations of the study: Only three studies investigated the possible role of MPs as biomarkers of the CD. The first article reports that increased circulating MPs could be linked to the type of inflammatory response underlying CD although the amounts of MPs do not correlate with disease activity [29]. It is important to note that only pro-coagulant MPs (annexin V^+^) were explored with the technique used. The cellular origin of these pro-coagulant MPs was mainly from platelets with low amounts from leukocytes. The second study measured only MPs from platelets and demonstrates that they are elevated in active but not inactive CD and correlate with disease activity and soluble P-selectin, suggesting that platelet MPs may be useful markers to evaluate activated state of platelets in this disease [30]. A third study published recently, reported a significant increase in tissue factor exposing MPs in IBD patients, but even their main objective was to explore their association to haemostasis activation, they also studied association to disease activity and CRP concentrations but found no significant correlations [44]. The present study highlighted a significant correlation between total levels of MPs, those from platelets and endothelial cells, and Harvey-Bradshaw index in a small cohort of patients. Altogether, although interesting, the use of MPs as biomarkers of CD needs to be explored on a much larger patients’ cohort, on longitudinal basis and a more detailed characterization of discriminated proteins and/or miRNAs carried by MPs.

In conclusion, the present study demonstrates that CDMPs should be considered as important factors in CD pathophysiology. It is not known if the induced endothelial and vascular dysfunction plays a role at the early stages of the disease and/or later. But the alterations induced by CDMPs, undoubtedly play a role in long-term intestinal damage and probably explain – at least in part – the vascular anatomical and functional abnormalities described several decades ago. Moreover, MPs not only should be considered as triggers of endothelial dysfunction, but also as effectors (i) able to amplify pre-existing vascular dysfunction, including vascular hypo-reactivity, (ii) possibly able to increase the prothrombotic status reported in CD, (iii) able to participate to the maintenance of chronic (even sub-clinical) inflammation, or (iv) contributing to extra-intestinal CD manifestations due to their ability to reach easily other organs than the gut.
